# Sivelestat improves acute lung injury by inhibiting PI3K/AKT/mTOR signaling pathway

**DOI:** 10.1371/journal.pone.0302721

**Published:** 2024-06-27

**Authors:** Yaqing Zhou, Haiyan Wang, Aiming Liu, Zunguo Pu, Qiuxia Ji, Jianhua Xu, Yuehua Xu, Ying Wang

**Affiliations:** 1 Department of Critical Care Medicine, Hai’an People’s Hospital, Hai’an County, Nantong City, Jiangsu Province, China; 2 Department of Critical Care Medicine, Hai’an Hospital of Traditional Chinese Medicine Affiliated to Nanjing University of Chinese Medicine, Nantong, Jiangsu Province, China; 3 Department of Clinical Pharmacy, Hai’an People’s Hospital, Hai’an County, Nantong City, Jiangsu Province, China; 4 Department of Critical Care Medicine, Nantong First People’s Hospital, Nantong City, Jiangsu Province, China; Pennsylvania State University Hershey Medical Center, UNITED STATES

## Abstract

**Objective:**

To investigate the therapeutic effect and mechanism of sivelestat sodium on acute lung injury (AIL).

**Methods:**

A rat model for ALI/acute respiratory distress syndrome (ALI/ARDS) was established. Pathological examination of lung tissue was conducted to assess lung injury. Blood gas in the arteries was measured using a blood analyzer. Changes in PaO_2_, PaO_2_/FiO_2_, and lung wet/dry (W/D) weight ratio were carefully compared. ELISA assay was conducted to estimate cell adhesion and inflammation response. Finally, real-time reverse transcription polymerase chain reaction and western blotting assay was used to determine the activation of PI3K/AKT/mTOR pathway.

**Results:**

ARDS in vivo model was successfully constructed by LPS injection. Compared with the sham group, PaO_2_ and PaO_2_/FiO_2_ were significantly lower in the vehicle group, while the lung W/D ratio, the lung injury score, NE, VCAM-1, IL-8 andTNF-αwere significantly increased. After treatment with different doses of sivelestat sodium, we found PaO_2_, PaO_2_/FiO_2_ were prominently increased, while the lung W/D ratio, the lung injury score, NE, VCAM-1, IL-8, TNF-α levels were decreased in the dose-dependent manner. Meanwhile, compared with the vehicle group, the expression levels of Bax, PI3K, Akt and mTOR were significantly lower, and the expression of Bcl-2 was significantly higher after injection with sivelestat sodium.

**Conclusion:**

Sivelestat sodium has an interventional effect on ALI in sepsis by inhibiting the PI3K/AKT/mTOR signalling pathway.

## 1. Introduction

Acute respiratory distress syndrome (ARDS) is one of the most lethal diseases for critically ill patients. It occurs in 10% of patients in the intensive care unit, with a mortality rate of 35%‒46% [1, 2]. Moreover, ARDS considerably contributes to the death of clinical patients with novel coronavirus disease (COVID-19), an ongoing global pandemic. Acute lung injury (ALI) and ARDS are characterized by cascade-amplifying, waterfall-like inflammatory secondary injury and secondary diffuse lung parenchymal injury. These injuries are caused by various inflammatory mediators and effector cells [3–5]. ALI and ARDS belong to the category of acute respiratory failure. Moreover, their etiology and pathogenesis are complex. Owing to numerous pathogenic links and a high fatality rate, ALI and ARDS have become prominent and challenging subjects in clinical intensive care research [5–7].

Current therapeutic strategies against ARDS mainly include mechanical ventilation and fluid administration. Drug therapy mainly includes anti-inflammatory drugs, nitric oxide, and beta-receptor agonists [8]. A study reported that of 29,144 patients admitted to the intensive care unit, 10. 4% received mechanical ventilation, and 23.4% of the patients had ARDS [9]. A meta-analysis reported that the efficacy of inhaled nitric oxide in the treatment of ARDS was associated with improved oxygenation. However, mortality did not reduce, prognostic indicators did not improve, and there was an increased risk of renal insufficiency [10]. Clinical studies have shown that β2-receptor agonists activate chloride and epithelial sodium channels, accelerate the transport of chloride and sodium ions, and accelerate the reduction of pulmonary edema [9–12]. Additionally, observational studies of other viral diseases suggested that corticosteroids might increase viral load in patients with SARS-CoV-2 and Middle East respiratory syndrome [13]. High-dose dexamethasone rapidly improves the clinical status and decreases inflammatory biomarkers, whereas CVD is increased [14]. Sivelestat treatment may improve the oxygenation index in patients with ARDS; however, it does not reduce short-term mortality [12]. Moreover, the efficacy and mechanism of sivelestat remain unclear.

Sivelestat sodium, a neutrophil elastase (NE) inhibitor, is a synthetic heterocyclic compound used for the clinical treatment of ARDS. Excess activity of NE and similar proteases causes tissue damage and alters the remodeling process in many clinical conditions, such as pneumonia, respiratory distress, and ALI [15]. Sivelestat sodium can effectively ameliorate the symptoms of ARDS, improve oxygenation levels, and shorten the duration required for mechanical ventilation and hospitalization [12, 16, 17]. However, the detailed therapeutic mechanism of sivelestat sodium against ARDS warrants further research. Consequently, sivelestat sodium is rarely adopted for clinical use.

In this study, lipopolysaccharides (LPS) were used to induce ARDS in rats. The rats were intraperitoneally (i.p.) administered sivelestat sodium, and their behavior was evaluated. Next, we evaluated the therapeutic efficacy of sivelestat sodium against ALI by measuring the contents of serum NE, VCAM-1, ICAM-1, IL-8, and TNF-α. Finally, we employed reverse transcription polymerase chain reaction (RT-PCR) and western Blot to quantify the expression levels in the PI3K/AKT signaling pathway, including PI3K, AKT, mTOR, Bax, and Bcl-2. There are few relevant studies to elaborate on the working mechanism of sivelestat sodium. This article provides the therapeutic role of the possible theoretical basis from a new perspective for sivelestat sodium treatment of acute lung injury.

## 2. Materials and methods

### 2.1 Animals

50 Sprague-Dawley (SD) male rats weighing 200 ± 20 g, were provided by the Experimental Animal Centre of Nanjing Medical University, Laboratory Animal Use License: SYXK (Su) 2020–0022. All rats were housed in the Experimental Animal Centre of Nanjing Medical University, with 5 rats/cage, maintained on feed-acclimatized diets for 5 days, with a 12-h light-dark cycle, free. All rats were kept at (25.0 ± 1.0°C and (50.0 ± 10.0)% relative humidity for 5 days with maintenance feed acclimatization, 12 h light/dark cycle, and free access to food and water. All experimental procedures complied with the guidelines for animal experiments set by Nanjing Medical University were approved by the Animal Ethics Committee and were in accordance with the ARRIVE guidelines. This study was approved by the Committee on the Ethics of Animal Experiments of Nanjing Medical University (IACUC2209020); the protocols followed the SOP specification. We respected and treated experimental animals well, maintained their welfare and ethics, followed the “3R” principle, and used them in a scientific and standardized manner. The handling and disposal of experimental animals shall comply with the relevant provisions of the “Guiding Opinions on Good Treatment of Experimental Animals” issued by the Ministry of Science and Technology of the People’s Republic of China in 2006.

### 2.2 Reagents and equipment

Sivelestat sodium was purchased from Shanghai Huilun Jiangsu Pharmaceutical Co., Ltd. The NE, VCAM-1, ICAM-1, IL-8, and TNF-α ELISA kits were bought from Nanjing Jiancheng Bioengineering Institute Co., Ltd. Additionally, the RNA extraction, cDNA synthesis, and fluorescence quantification kits were bought from Nanjing Jiancheng Bioengineering Institute Co., Ltd. The pre-staining Marker was purchased from Thermo, USA; RNA extraction kit, cDNA synthesis kit, and fluorescence quantification kit were purchased from Nanjing Jianjian Bioengineering Institute Co. The Victor TM X3 microplate reader was purchased from PerkinElmer (USA). The TDZ5-WS centrifuge was bought from Hunan Xiangyi Centrifuge Instrument Co., Ltd. A BSA224S analytical balance was purchased from Sartorius, Germany. The 7500/Bio-RAD iCycler real-time quantitative PCR instrument was bought from BioRad, USA. Finally, an i-STAT clinical blood analyzer was bought from ABBOTT, USA.

### 2.3 Experimental methods

#### 2.3.1 *In vivo* studies

Healthy male SD rats were randomly divided into 5 groups (10 rats per group): blank (sham), ARDS modle(vehicle), and high-, medium-, or low-dose sivelestat sodium. Rats in the sham group were i.p. injected with saline (dose: 2 mL/kg). Those in the other 4 groups were first i.p. injected with LPS (dose: 4 mg/kg) to establish ARDS models. After 1 h, these rats were i.p. injected with saline (vehicle group) or different doses (6, 10, or 15 mg/kg, calculated according to the manufacturer’s instruction and converted into the equivalent dose for humans/rats) of sivelestat sodium [18]. The behavior of the rats in these groups was observed. Subsequently, PaO_2_ and PaO_2_/FiO_2_ were measured to confirm the successful establishment of the ARDS model.

#### 2.3.2. PaO2/FiO2 ratio measurement

Rats were i.p. anesthetized using pentobarbital injections. Arterial blood samples were obtained from the carotid artery. Next, PaO_2_ analysis was conducted on the arterial blood samples using a GEM Premier 3,500 blood gas analyzer (Instrumentation Laboratory Co, Mass). The oxygenation index was expressed as the arterial partial pressure of oxygen/fraction of inspired oxygen.

#### 2.3.3 Measurement of lung wet/dry (W/D) weight ratio

Treatment with sivelestat sodium for 5 days. 6 h after the last post-injection, we were subject to blood collection from their abdominal aorta. The blood samples were stored at 4°C for 1 h, followed by centrifugation at 3500 rpm for 15 min. Then, the supernatants were collected for further use. 30 min later, the rats in each group were anesthetized with 0.4% pentobarbital sodium solution (40 mg/kg, i.p.) and painlessly sacrificed by bloodletting from the right femoral arter, and their lungs were excised from the thoracic cavity. Next, the blood was quickly wiped from the surface with filter paper. The whole lung tissue was then weighed on an electronic balance and referred to as lung tissue wet weight (W). The whole weighed lung tissue was baked at 70°C for 72 h at a time to maintain a constant weight. Then, the lung was placed on filter paper and weighed on an electronic balance. This was subsequently referred to as lung tissue dry weight (D). Finally, 100 mg of the lung tissue in each group was collected and stored at -80°C for further use.

#### 2.3.4 Biochemical analysis of serum

The serum samples of rats in each group were collected (shown in 2.2), and the levels of NE, VCAM-1, ICAM-1, IL-8, and TNF-α were measured using corresponding ELISA kits according to the manufacturer’s protocol.

#### 2.3.5 Histopathological examination of the lungs

The right lung tissue was isolated from each group of rats, and the remaining blood was washed with saline, then perfused with “1% formalin + 0.5% agarose” at a pressure of 20 cm H_2_O, and the same part of the tissue block was fixed with 10% neutral formaldehyde solution for 1 day, then embedded in paraffin wax, serially sectioned(5μm), and stained with hematoxylin-eosin (HE). Five indexes of neutrophils in the alveolar space, neutrophils in the interstitial space, hyaline membranes, proteinaceous debris and alveolar septal thickeningn were observed. Lung injury was scored according to the American Thoracic Society workshop report [19].

#### 2.3.6 RT-PCR analysis

The lung tissues of rats in each group were collected to extract total RNA using the TRIzol method. The RNA concentration was measured using ultraviolet-visible spectroscopy. Next, the reaction system was prepared using a commercial reverse transcription kit to obtain cDNA. The primers used were synthesized by Shanghai Sangon Biotech Co., Ltd., and their sequences are listed in Table 1. RT-qPCR was used to measure the Ct value of each sample under the following reaction conditions: 95°C for 5 min, 95°C for 30 s, 60°C for 30 s, and 72°C for 30 s; 40 total recycles. After the reaction, the relative expression levels of Bax, Bcl-2, PI3K, Akt, and mTOR were calculated through the 2^-ΔΔCt^ method using β-actin as an internal control.

**Table 1:** Primers for RT-qPCR.

Gene	Upstream primer sequence	Downstream primer sequence	Product length (bp)
	(5’→3’)	(5’→3’)	
Bax	GGACGCATCCACCAAGAAG	CTGCCACACGGAAGAAGAC	134 bp
Bcl-2	GGGATGCCTTTGTGGAAC	GTCTGCTGACCTCACTTG	148 bp
PI3K	GCAGTTTTGGAAGCAGTCACA	ATTCAGTTCAATTGCAGAAGGAG	233 bp
Akt	ACACCAGGTATTTTGATGAGGAG	TCAGGCCGTGCCGCTGGCCGAGTAG	143 bp
mTOR	GCAACCCTTCTTTGACAACATTTTT	ATTTCTTCTCTCAGACGCTCTCC	297 bp
β-actin	GACAAGATGGTGAAGGTCGG	CTGGAAGATGGTGATGGGTTT	229 bp

#### 2.3.7 Western bloting

The tissues were removed from -80°C refrigerator, weighed 100 mg rat tissues, placed in 1.5 ml EP, added 1 ml protein lysate, subjected to SDS-PAGE gel electrophoresis, transferred to the membrane, closed in 5% skimmed milk, incubated with primary antibodies (Bax, 1:1000; Bcl-2, 1:1000; PI3K, 1:800; Akt. 1:1000; mTOR, 1:1000, β-actin, 1:1000), and the PVDF membrane was scanned and developed by applying Odyssey Infrared Imaging System to analyze the grey value of the target bands, using β-actin as an internal reference. The grey scale of the target protein was compared with that of β-actin, and the resulting ratio was the relative expression of the target protein.

### 2.4 Statistical analysis

Statistical data were analyzed using the SPSS 20.0 software. Quantitative data were analyzed by mean ± standard deviation, and paired F test was used to compare data between multiple groups. Measurement data of more than two groups were analysed by ANOVA. If they conformed to normal distribution, a rank sum test was used if they did not conform. p<0.05 was statistically different.

## 3. Results

### 3.1 Behavioral changes

Healthy rats in the sham group were in good clinical condition, as characterized by their glossy hair, red lips and extremities, and the absence of foam-like liquid in the mouth and nose. In contrast, the treated rats presented higher respiratory frequency and other abnormal symptoms, such as wheezing, cyanosis, hair raising, and anorexia after intraperitoneal injection of LPS. A proportion of the LPS-treated rats had foam-like liquid flowing from their mouths and noses. The physiological condition of the rats was exacerbated from irritability to malaise with time, with obvious drowsiness. In addition, the response to external stimuli was weakened. Following the administration of different doses of sivelestat sodium, the respiratory rate of the rats decreased, the breathing was smooth, the color of the limbs gradually recovered, and there was no bloody foamy liquid flowing from the mouth and nasal cavity. This was particularly evident in the high-dose group.

### 3.2 Changes in blood gas parameters

PaO_2_ and PaO_2_/FiO_2_ represent two important parameters reflecting the degree of ARDS. As shown in Table 2, the PaO_2_ and PaO_2_/FiO_2_ values of the vehicle group were significantly lower than those in the sham group (*P* < 0.01). In contrast, the values of PaO_2_ and PaO_2_/FiO_2_ of the sivelestat sodium groups exhibited an increasing trend. PaO_2_ and PaO_2_/FiO_2_ values positively correlated with dosage, especially in the medium- and high-dosage groups having high PaO_2_ and PaO_2_/FiO_2_ values.

**Table 2:** Values of blood gas indices, W/D weight ratios and lung injury score (n = 10;$\bar{\text{x}}$±s).

Group	Dose (mg/kg)	PaO_2_ (mmHg)	PaO_2_/FiO_2_	W/D (g/g)	Lung injury score
Sham group	-	88.15 ± 4.74	426.21 ± 7.11	4.09 ± 0.17	0.1 ± 0.1
Vehicle group	4	48.99 ± 4.99**	284.97 ± 6.43**	8.28 ± 0.24**	0.82 ± 0.20**
High-dose group	15	80.87 ± 4.87^##^	386.87 ± 6.35^##^	6.79 ± 0.18^#^	0.54 ± 0.13^##^
Medium-dose group	10	71.36 ± 4.56^##^	344.17 ± 6.29^#^	7.15 ± 0.20	0.70 ± 0.14
Low-dose group	6	61.93 ± 4.47^#^	306.35 ± 6.14	7.74 ± 0.21	0.74 ± 0.13

*: compared between the sham and vehicle group, *P < 0.05, **P < 0.01; #: compared between the vehicle group and sivelestat sodium groups: ^#^*P* < 0.05, ^##^*P* < 0.01.

### 3.3 Histological observation

**Fig 1:**
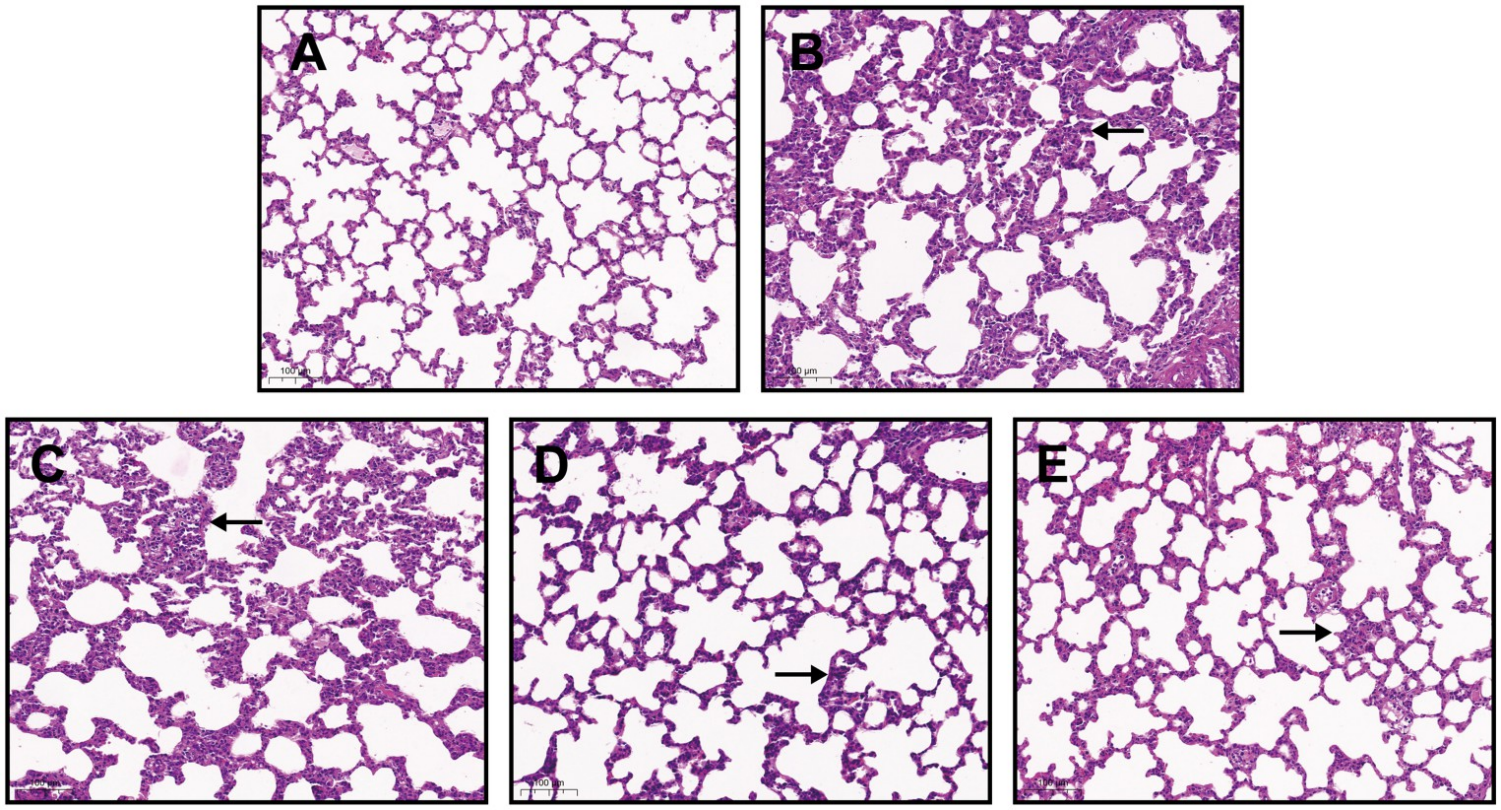
Comparison of pathological characteristics among groups (supporting information files can be found in S1 Raw images). A: sham group, B: vehicle group, C: low-dose group, D: medium-dose group, E: high-dose group. The lung tissue structure of rats in the sham group was clear, with thin and smooth alveolar walls, consistent alveolar intervals, and no invasion of inflammatory cells; in the vehicle group the alveolar spaces were narrowed or disappeared, with significant increase of inflammatory cells in the lumens, lung congestion, interstitial oedema, and diffuse inflammatory cell infiltration. Administration of sivelestat sodium resulted in a slight narrowing of the alveolar spaces, thickening of some alveolar lumens, lung congestion, mild interstitial oedema, and small focal inflammatory cell infiltration, with the improvement being particularly marked in the high-dose group.

We sectioned the lungs of rats and observed them under a microscope as shown in Fig 1.

### 3.4 Changes in lung W/D weight ratios and lung injury score

The W/D weight ratio of the lung tissue serves as a crucial indicator of lung permeability. As shown in Table 2, Compared with the sham group, the lung W/D ratio and lung injury score of rats in the vehicle group were significantly higher than those in the sham group (P<0.01); compared with the vehicle group, the lung W/D ratio and lung injury score of rats in the high dose group of sivelestat sodium were significantly lower (P<0.05 or P<0.01).

### 3.5 Changes in serum NE, VCAM-1, ICAM-1, IL-8, and TNF-α levels

The levels of serum NE, VCAM-1, ICAM-1, IL-8, and TNF-α from rats in different groups are listed in Table 3. The LPS-treated group exhibited significantly higher levels of serum NE, VCAM-1, ICAM-1, IL-8, and TNF-α than the sham group (*P* < 0.01). The high- (*P* < 0.01) and medium-dose (*P* < 0.05 or 0.01) sivelestat sodium groups exhibited significantly lower serum NE, VCAM-1, ICAM-1, IL-8, and TNF-α levels than the LPS-treated group.

**Table 3:** Values of indicated biochemical indices (n = 10; $\bar{\text{x}}$± s).

Group	NE (ng/mL)	VCAM-1 (ng/mL)	ICAM-1 (ng/mL)	IL-8 (pg/mL)	TNF-α (ng/mL)
Sham group	7.20 ± 0.04	4.18 ± 0.73	197.46 ± 33.79	515.04 ± 35.57	79.15 ± 1.28
Vehicle group	10.19 ± 0.06**	19.63 ± 2.61**	287.65 ± 37.31**	1594.17 ± 56.77**	119.51 ± 2.82**
High-dose group	8.14 ± 0.05^##^	10.61 ± 2.36^##^	237.23 ± 36.11^##^	813.79 ± 46.35^##^	84.56 ± 2.36^##^
Medium-dose group	8.98 ± 0.06^#^	12.17 ± 2.42^#^	255.98 ± 36.51	1032.56 ± 50.16^##^	92.36 ± 2.41^#^
Low-dose group	9.24 ± 0.07	16.82 ± 2.46	269.69 ± 36.82	1345.86 ± 54.67	106.88 ± 2.29

*: compared between the sham and vehicle group, **P < 0.01; #: compared between the vehicle group and sivelestat sodium groups: ^#^*P* < 0.05, ^##^*P* < 0.01.

### 3.6 Bax, Bcl-2, PI3K, Akt, P-Akt and mTOR protein expression in lung tissues of rats of each group

**Fig 2:**
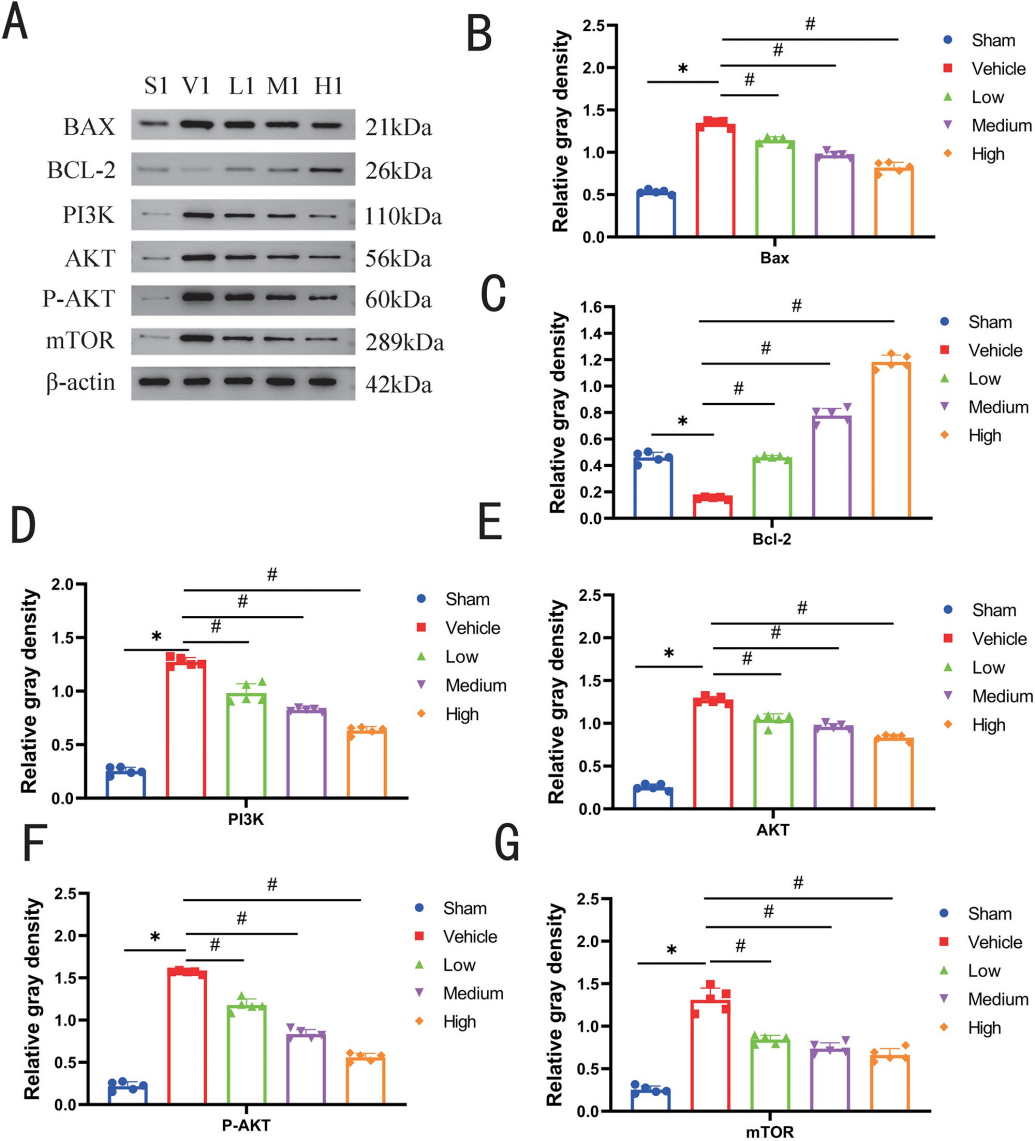
Changes in relative protein expression of Bax, Bcl-2, PI3K, Akt, P-Akt and mTOR in the lung tissues of rats in each group (supporting information files can be found in S2 Raw images). Compared with the sham group, the expression of Bax, PI3K, Akt, P-Akt and mTOR proteins in the vehicle group was significantly higher (P<0.05), and the expression of Bcl-2 proteins was significantly lower (P<0.01); compared with the vehicle group, the expression levels of Bax, PI3K, Akt, P-Akt and mTOR proteins in the lung tissues of the rats of each dose group of sivelestat sodium were significantly lower (P<0.01). Akt and mTOR protein expression levels were significantly reduced (P < 0.05) and Bcl-2 protein expression was significantly increased (P < 0.01) in the lung tissues of rats in each dose group of sivelestat sodium. Note: *: compared between the sham and vehicle group, *P < 0.05; #: compared between the vehicle group and sivelestat sodium groups: ^#^*P* < 0.05.

The results of the proteins in the lung tissues of rats in each group are shown in Fig 2.

## 4. Discussion

Sepsis is a life-threatening organ dysfunction caused by a dysregulation of the body’s immune response to infection, and there were approximately 11 million sepsis-related deaths in 2017 [20]. Sepsis is a common cause of acute lung injury in critically ill patients, and the pathology is characterised by infiltration of granulocytes in the lungs, which induces interstitial and intra-alveolar oedema, with surfactant depletion and formation of atelectasis, and the mortality rate is approximately 40% to 60% in severe ARDS [21].

### 4.1 Therapeutic effect of sevilastat sodium on ALI in sepsis

Sivelestat sodium, as a new type of drug for ALI, can inhibit neutrophil elastase (NE) activity, and is an effective drug for the treatment of acute lung injury or acute respiratory distress syndrome with systemic inflammatory response syndrome. In recent years, clinical studies have shown that it can effectively improve the symptoms of ALI, improve oxygenation levels, and reduce the duration of mechanical ventilation and hospital stay in patients [17], and sivelestat sodium may be a potentially effective drug for the treatment of novel coronavirus pneumonia-induced ALI or disseminated intravascular coagulation. In the present study, sivelestat sodium was applied to treat LPS-induced septic lung injury in rats, and by observing the behavioural and blood gas analysis of the rats, PaO_2_ and PaO_2_/FiO_2_ were significantly decreased in the vehicle group compared with the sham group (P<0.01), and PaO_2_ and PaO_2_/FiO_2_ were significantly increased after administration of the drug (P<0.05 or 0.01).

The lung tissue of acute lung injury in sepsis is invaded by a large number of inflammatory cells, resulting in microenvironmental disorders in vivo, thickening of alveolar structures, and exacerbation of lung wet weight/dry weight [22]. The results of this study showed that the administration of sivelestat sodium resulted in slightly narrowed alveolar spaces, thickening of some alveolar lumens, congestion of the lungs, mild pulmonary interstitial oedema, and small focal inflammatory cell infiltration, with the improvement being particularly obvious in the high-dose group. Compared with the sham group (S1 Raw images), the lung W/D ratio and lung injury score of rats in the vehicle group were significantly higher than those in the normal sham group (P<0.01); compared with thevehicle group, the lung W/D ratio and lung injury score of rats in the high dose group of sivelestat sodium were significantly reduced (P<0.05 or P<0.01). This indicates that it was able to confirm the ameliorative effect of sivelestat sodium in improving acute lung injury in rats with sepsis.

### 4.2 Sivelestat sodium and the inflammatory response in ALI in sepsis

NE is a potential mucinous secretagogue for mucinous gland cells and goblet cells, which can increase mucin synthesis and mucus secretion. NE is a terminal effector factor that causes the inflammatory cascade of ALI, mainly involved in and initiating the occurrence of ALI/ARDS by damaging capillary endothelial cells and alveolar epithelial cells, digesting and degrading extracellular matrix (ECM), and epithelial junction structures [23]. In addition, NE can upregulate the expression of VACM-1 and ICAM-1 and promote adhesion between neutrophils and endothelial cells. This study shows that compared with the sham group, the vehicle group has an increase in serum NE, VACM-1, and ICAM-1 levels. After intraperitoneal injection of sivelestat sodium, the levels of the three significantly decreased, indicating that sivelestat sodium can inhibit the upregulation of NE, inhibit the expression of VACM-1 and ICAM-1, and prevent the production of ARDS.

In terms of the expression of inflammatory factors, NE can induce epithelial cells to release various pro-inflammatory cytokines such as IL-6 and IL-8, increasing the intensity of inflammatory response. On the other hand, NE can affect the adhesion and migration of neutrophils and degrade various pro-inflammatory cytokines such as TNF-α And pro-inflammatory cytokines such as IL-2 inhibit the process of inflammatory response, weaken the adhesion and migration of T cells, affect the body’s immunity, and regulate inflammatory response [24, 25]. This study shows that compared to the sham group, the serum levels of IL-8 and TNF-α in the vehicle group were obviously increased. After intraperitoneal injection of sivelestat sodium, the levels of both significantly decreased, indicating that sivelestat sodium can inhibit IL-8 and TNF by regulating NE- α The expression of inflammatory factors can prevent the production of ARDS.

### 4.3 Regulation of the PI3K/AKT/mTOR signalling pathway by sivelestat sodium in acute lung injury in sepsis

Sivelestat sodium can exert pulmonary protection through various pathways. In animal studies of acute severe pancreatitis, sivelestat sodium exerted pulmonary protection by inhibiting the signal transducer and activator of transcription (STAT) or TNF-α pathway [26], and in septic mice administered sivelestat sodium inhibited sepsis-induced macrophage infiltration, overproduction of pro-inflammatory mediators, and activation of the serine/threonine kinase pathway, which attenuated the renal injury induced by sepsis, and exerted a certain degree of nephroprotective effect [27].

NE activates the TRL4 receptor, which further upregulates the expression and synthesis of nuclear transcription factor-κB (NF-κB). trl4 receptor activation first activates the PI3K protein, and activated PI3K prompts the conversion of PIP2 to PIP3, which further binds to the PH structural domain-containing signalling protein AKT and activates phosphorylated AKT. p-AKT then inhibits the downstream Bcl-2, Caspase-9, Caspase-3 and other molecules to induce apoptosis and inflammatory responses [3, 28]. In addition p-AKT is able to activate downstream mTOR and NF-κB molecules involved in promoting cell proliferation, growth, and survival. ji et al. demonstrated that the use of the PI3K inhibitor, wortmann penicillin, in lung tissues of mice with ARDS up-regulated the level of p-AKT protein expression and exacerbated ARDS and lung injury [29]. This shows that the PI3K/AKT signalling pathway plays an important role in ARDS. In this study, we showed that the expression of PI3K, AKT, mTOR, and Bax was elevated, while Bcl-2 expression was decreased in the ARDS model. After intraperitoneal injection of sivelestat sodium, the expression of PI3K, AKT, mTOR, and Bax was decreased, while the expression of Bcl-2 was increased (S2 Raw images). It can be speculated that NE can activate the PI3K/AKT/mTOR signalling pathway, up-regulate VACM-1 and ICAM-1 to promote neutrophil-endothelial cell adhesion and induce ARDS, and lead to the release of downstream pro-inflammatory factors and inhibition of anti-inflammatory factors. After intraperitoneal injection of sivelestat sodium, the expression of PI3K, AKT, mTOR, and Bax was significantly reduced, and the expression of Bcl-2 was elevated, while the levels of VCAM-1 and ICAM-1 were down-regulated. It is thus hypothesized that sivelestat sodium can improve the lungs and the related inflammatory responses by inhibiting apoptosis and the release of inflammatory factors, and thus exerts its protective effect on lung injury in ARDS rats.

The limitation of this study is that we only verified the efficacy of sivelestat sodium using in vivo experiments. We will select alveolar epithelial cells to further verify the effect and mechanism of sivelestat sodium in vitro.

In conclusion, sivelestat sodium acts as an effective clinical drug for the treatment of ARDS in animal models by inhibiting the release of apoptotic and inflammatory factors via the PI3K/AKT/mTOR signaling pathway. This study provides both experimental evidence and a mechanistic basis for the efficacy of sivelestat sodium in LPS-induced ARDS in animal models. We have shown for the first time the mechanism of sivelestat sodium action from this point of view, providing a theoretical basis for clinicians to apply sivelestat sodium.

## Supporting information

S1 Raw images Original H&E images for Fig 1. (TIF)

S2 Raw images Original image of blot for Fig 2. (TIF)

S1 File (PDF)
