# Supplementary figures and images for: Sivelestat improves acute lung injury by inhibiting PI3K/AKT/mTOR signaling pathway
Source: PLoS One. 2024 Jun 27;19(6):e0302721. doi: 10.1371/journal.pone.0302721 (PMC11210789; doi:10.1371/journal.pone.0302721)

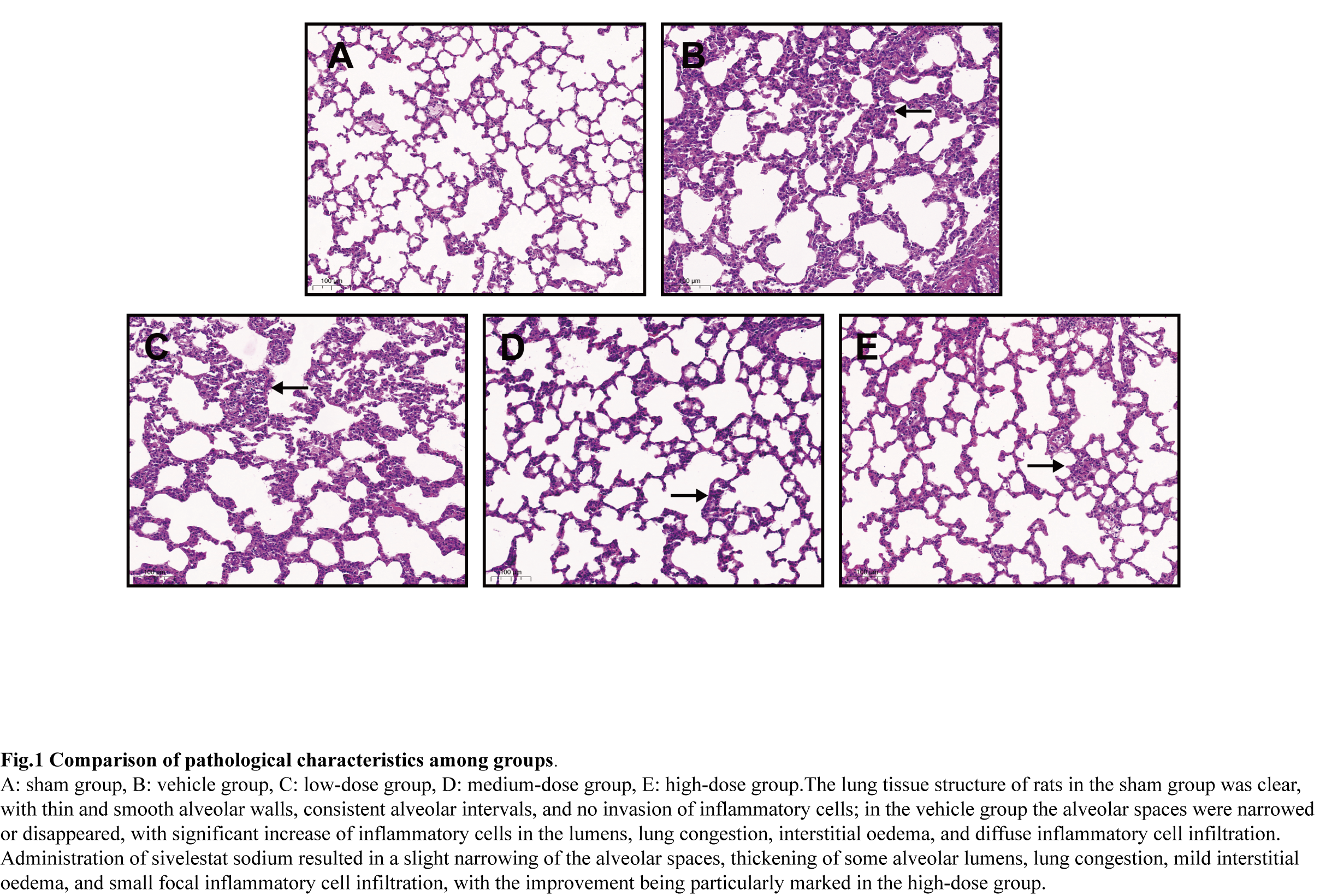

Supplement: S1 Raw images — (TIF) [file pone.0302721.s001.tif]

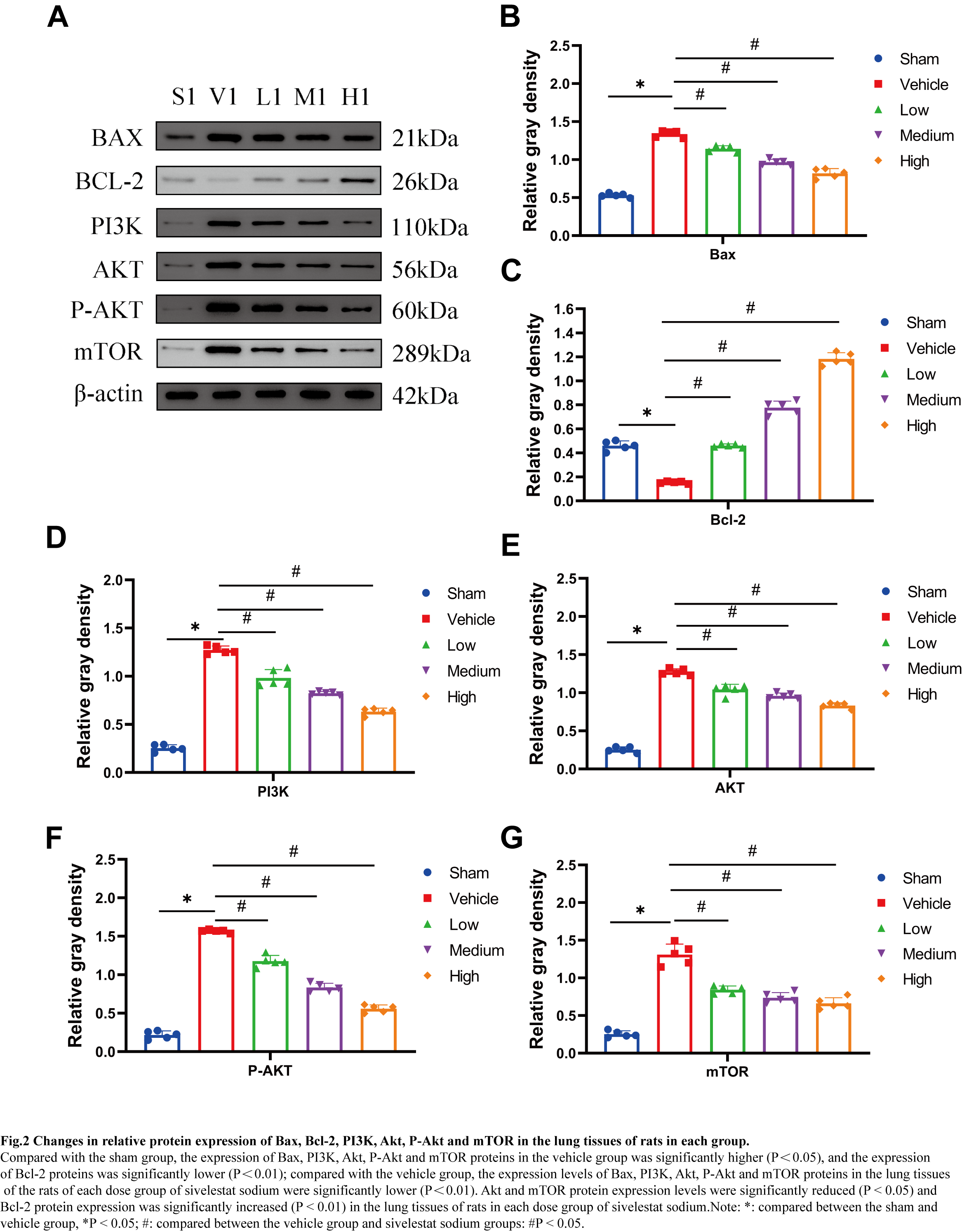

Supplement: S2 Raw images — (TIF) [file pone.0302721.s002.tif]

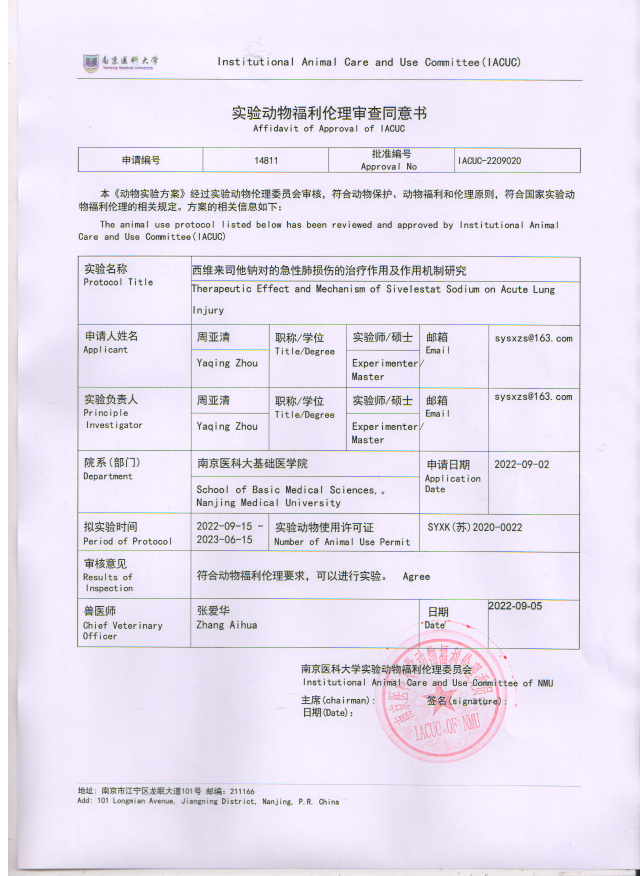

Supplement: S1 File — (PDF) [file pone.0302721.s003.bmp]
